# Assistive Technology Provider Experiences during the COVID-19 Pandemic

**DOI:** 10.3390/ijerph181910477

**Published:** 2021-10-06

**Authors:** Louise Puli, Natasha Layton, Daniel Mont, Kylie Shae, Irene Calvo, Keith D. Hill, Libby Callaway, Emma Tebbutt, Abner Manlapaz, Inge Groenewegen, Diana Hiscock

**Affiliations:** 1 Access to Assistive Technology Team, World Health Organization, 1211 Geneva, Switzerland; shaek@who.int (K.S.); calvoi@who.int (I.C.); tebbutte@who.int (E.T.); 2 Rehabilitation, Ageing and Independent Living Research Centre, Monash University Australia, Clayton 3800, Australia; natasha.layton@monash.edu (N.L.); keith.hill@monash.edu (K.D.H.); libby.callaway@monash.edu (L.C.); 3 Center for Inclusive Policy, Washington, DC 20005, USA; daniel.mont@inclusive-policy.org (D.M.); abner.manlapaz@inclusive-policy.org (A.M.); 4 Occupational Therapy Department, Monash University Australia, Clayton 3800, Australia; 5 Liliane Fonds, 5211 TX s-Hertogenbosch, The Netherlands; IGroenewegen@lilianefonds.nl; 6 HelpAge International, London SE1 7RL, UK; diana.hiscock@helpage.org

**Keywords:** assistive technology, policy, disability, aging, assistive products, personnel, service provision, health systems, COVID-19

## Abstract

Globally, health systems face challenges in the delivery of assistive technology (AT) and only 10% of people are currently able to access the assistive products they need. The COVID-19 pandemic presented an uncharted path for AT providers to navigate, placing them under pressure to be agile and rapidly adapt. This article, part of a series, explores the experiences and impacts of the COVID-19 pandemic on AT providers and aims to inform how AT providers can be better prepared and supported in the future. A mixed methods approach was used to gather service data and perspectives from AT providers via a survey. A total of 37 responses were received from 18 countries. Service data showed extensive service disruption throughout 2020. Thematic analysis suggested significant changes to routine AT service delivery including rapid momentum towards home-based, decentralised, and digital services for which many AT providers were not prepared. Providers were required to make difficult decisions and deliver services in new ways to balance meeting demands, complying with government restrictions, and ensuring the safety of staff and clients. Few but important positives were expressed including the belief that expanded capacity to use remote and digital AT service delivery would remain useful in the future.

## 1. Introduction

The World Health Organization (WHO) declared the coronavirus disease (COVID-19) a global pandemic in March 2020, prompting an unprecedented public health response and impacting health systems and services [1]. Untested public protection mechanisms were rapidly implemented to reduce transmission as governments released new national laws and policies, and guidance was given by international bodies such as the WHO. Providers of non-COVID-19-related health services are impacted by public health responses. For example, assistive technology (AT) services, when considered “non-essential” by governments, were forced to close, and restrictions on public movement limited the accessibility of AT services, which are often far from people’s homes. Such measures to curb the transmission of COVID-19 have had unintended consequences for AT users who were unable to access the services they need to receive or maintain their assistive products [2,3,4]. Consequently, providers of non-COVID-19-related health services such as AT had to be agile, adapting rapidly as policies and procedures changed.

AT is an umbrella term describing systems and services related to the provision of assistive products [5]. Assistive products enable people to function, participate and live more independently through improving their interaction with an activity or environment. Assistive products assist functioning, which can be categorised by the functional metrics of mobility, self-care, vision, hearing, cognition, and communication [6]. Common examples include hearing aids, wheelchairs, prostheses and orthoses, white canes, glasses, hearing aids and communication boards. The range of personnel who provide assistive products can be found at all levels of the health system and are often referred to as AT providers.

The pre-COVID-19 global population with a need for AT was estimated to be one billion people, projected to rise to two billion people by 2030 [7]. This need is now likely to be much greater for two reasons, firstly the emerging long-term impact of COVID-19 on those who contracted it [8] and secondly, the functional declines associated with the public health response to COVID-19, which prevented access to AT services for those with existing conditions [2]. AT is essential for many, and when not available or in the event that populations are prevented from accessing them, AT users may lose function, and reduce their participation in employment, education and in the community [9,10].

AT providers (personnel and managers of AT services) are accustomed to providing safe and effective services via standardised practice steps, which traditionally include single or multiple face to face contacts with the client. The steps used in the provision of AT have been organised under four key areas by the WHO, including selection (including screening and assessment), fitting and adjustment, user training, and follow up (including maintenance and repairs) [5]. Ongoing AT services include maintenance and repair for assistive products currently in use and adjustments to enhance function, comfort and fit. Disruption of any of these individual steps will impact AT user outcomes, as each is integral to a safe and effective AT service. Prior to COVID-19, community-level, outreach, or tele-AT services were extremely rare, meaning people residing in rural or remote areas commonly travel vast distances for their AT services [11].

The literature exploring the impact of COVID-19 on people living with disabilities and older persons, those likely to require regular access to AT, is steadily increasing [12,13,14,15,16,17,18,19,20,21]. The impact of COVID-19 on AT providers, however, has been less commonly investigated. Given that AT services are provided by a range of professions across a variety of healthcare and community settings, it is likely that not all AT providers were impacted equally or in the same way.

Considering the vital role of effective AT in the lives of those who use it, it is important to understand how AT providers have been impacted by COVID-19 and to what extent AT services adapted or changed. The impacts of COVID-19 and subsequent public health responses were not uniform; therefore, exploring AT provider experiences across a variety of settings and cultures may help to identify ways to build more resilient AT services and systems capable of operating within the context of a global health crisis. The objectives of this study therefore were to explore how global AT providers were impacted by and responded to the COVID-19 pandemic and the public health response. Using this evidence, the aim is to inform how AT providers can be better prepared and supported in the future by resilient AT policies and systems. This, in turn, will aid more effective outcomes for people using AT.

## 2. Materials and Methods

This mixed methods study was implemented in two phases. The first phase involved a rapid literature review utilising keywords related to AT, COVID-19, disability and ageing to establish existing and emerging themes. These identified themes formed the basis of the development of an accessible survey (Appendix A), which was drafted and piloted by the research team. The second phase involved dissemination of the survey to collect information about the experience of AT providers during the COVID-19 pandemic. 

Ethics approval was granted for this project by the Monash University Human Research Ethics Committee, HREC 26972 prior to data collection commencing. Formal ethical approval ensures research complies with Australia’s National Ethical Statement on the conduct of Human Research https://www.nhmrc.gov.au/about-us/publications/national-statement-ethical-conduct-human-research-2007-updated-2018 (accessed on 24 September 2021), which includes clear strategies to enshrine the voluntary nature of participation and to manage burden, for example, in the participant information statement.

### 2.1. The Research Team

The research team includes international representation with expertise in AT, policy and disability from academic institutions, international non-governmental organisations and from the WHO’s Assistive Technology Team. This global team allowed for the survey used for data collection to be piloted to ensure that it was clear in language and was accessible and culturally suitable across each of the six WHO world regions.

### 2.2. Literature Review and Survey Development

Given the speed of COVID-19-related additions to the literature, our rapid literature review sourced relevant peer-reviewed literature at two time points (October 2020; March 2021). Several iterations of keyword searches were trialled by two separate authors (NL and IC) (assistive products; older people; survey; disability; ageing) and the final search string was agreed upon (assistive technology and COVID-19) before it was run in PubMed and Google Scholar. Six hundred and sixty-two unique titles and abstracts were screened, with fifty-two full-text records reviewed and a final yield of forty studies providing a rapid review of the current COVID-19, disability, and AT literature. Four authors (NL, IC, LP, DM) reviewed the final list of identified studies and together drafted the survey questions. A first draft of the survey was shared with the research team who reviewed the included questions, language, format and cultural suitability. A meeting was held to hear regional perspectives and adjustments were made to the survey until consensus was reached. 

The literature review also informed the discussion and recommendations.

### 2.3. Participant Recruitment

Participants in this research were AT providers (personnel and service managers). The sample of participants were drawn from each of the six WHO Regions (Table 1). The research team used the WHO’s existing register of global networks to disseminate information about the research to sixty-nine government, private, academic, and training institutions, user group organisations, and non-governmental organisations. Contact was made with organisations in December 2020 via email, inviting AT providers to volunteer their participation and requesting them to pass on the invitation to other AT providers in their network. The email included attachments consisting of a Participant Information Statement, consent form and the survey form (Appendix A, Table A1). This snowball method encouraged dissemination out to AT providers known through networks external to our own. Recipients were invited to contact the principal investigator if they had difficulties filling in the survey or had any questions, or they could consent to participating by returning the signed consent form.

### 2.4. Survey

The survey was presented in Microsoft Word (Microsoft Corporation, North Sydney, Australia) in English and could be filled out using a computer or printed and filled out by hand. The survey contained 37 questions—28 closed ended questions and 9 open-ended questions. Firstly, participants were asked demographic questions including their location, the type of AT service they provide, and how their AT service is funded. Secondly, the months their AT service was impacted by COVID-19 between January 2020 and March 2021 were sought. Finally, participants were asked to provide the number of clients that attended their service in April, July and November 2019 and 2020 to serve as comparison.

Likert scales were used to seek AT providers’ perspectives about the level of difficulty (no difficulty, a little difficulty, a lot of difficulty, prevented service delivery, not applicable) caused by a variety of COVID-19-related impacts and also about what factors helped (none, a little, a lot, essential, not applicable). 

Open ended questions were placed after each Likert scale to allow respondents to expand and describe their experience, with no limits on the length of responses, and included:

1. Did your ability to provide products and services change?
2. What factors caused difficulty in your ability to provide AT services?
3. Did any other factors cause difficulty?
4. Did any other factors help you provide services?
5. What steps did you take to overcome the difficulties you have encountered?
6. What steps did other entities like the government or civil society organizations take to help you and was their help effective?
7. What is the biggest need of the users of your services and products currently?
8. What are the biggest needs for service providers to be able to meet the demand for your services and products?
9. What do you think could have been helpful, but was not done?

All survey data collected between 1 December 2020 and 5 March 2021 were included.

The sampling matrix classified respondents by: WHO World Region (African Region, Region of the Americas, Southeast Asian Region, European Region, Eastern Mediterranean Region, Western Pacific Region), and World Bank Income Classification (low income, low–middle income, upper–middle income, and high income). Service settings were considered (city, town/semi dense area, rural) and the presence of an outreach service was established. The types of AT provided by respondents were categorised into seven categories (cognition, communication, hearing, mobility, self-care, vision and AT support services).

### 2.5. Data Analysis

Quantitative data from returned surveys were manually entered into Microsoft Excel Office 365 ProPlus (Microsoft Corporation, North Sydney, Australia). Accuracy of data entry was confirmed through systematic line-by-line matching of paper and electronic versions by two authors (LP and NL). Respondent demographics were summarised using descriptive statistics appropriate to the data type including country, income level of the country and service setting. Quantitative data related to AT service attendance numbers and months where services were impacted were pooled and analysed to establish central tendencies of the data via the mean and percentages.

### 2.6. Qualitative Analysis

Qualitative data from the open-ended survey questions were uploaded into NVivo v.11 (QRS International, London) for analysis. 

**Figure 1:**
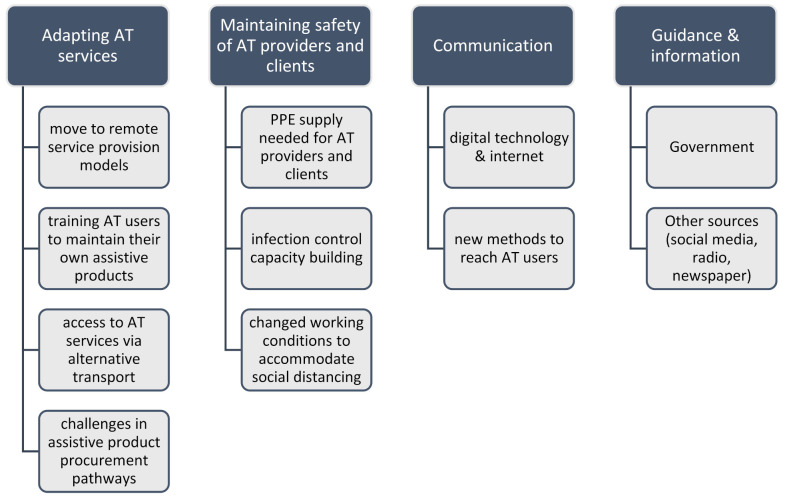
Thematic coding tree.

Two researchers (LP, NL) undertook independent line-by-line coding to desegregate survey text. Text describing similar phenomena was categorised into nodes from which themes and subthemes were developed. The themes and subthemes were presented to the whole research team once 50% of coding had taken place. A thematic coding tree (Figure 1) along with illustrative examples of first-person quotes were proposed. Themes were discussed sequentially and disagreements with the interpretation were resolved by further discussion until consensus was reached [22]. The final themes and subthemes are presented in the results narrative with illustrative first-person quotes to support and evidence the interpretation [23]. The qualitative narrative is arranged by the WHO’s model for AT systems strengthening, including the areas of policy, people, provision, products, and personnel (Section 3.6).

## 3. Results

### 3.1. Responses

From the 69 organisations requested to participate and disseminate the survey, responses were received from 37 AT providers from 18 countries with representation from each of the 6 WHO World Regions (Table 1). Low–middle-income settings comprised about half (48%) of the responses followed by high-income countries (22%). The Southeast Asian Region provided the most responses (32%) and the least responses were received from the European Region (3%).

**Figure 2:**
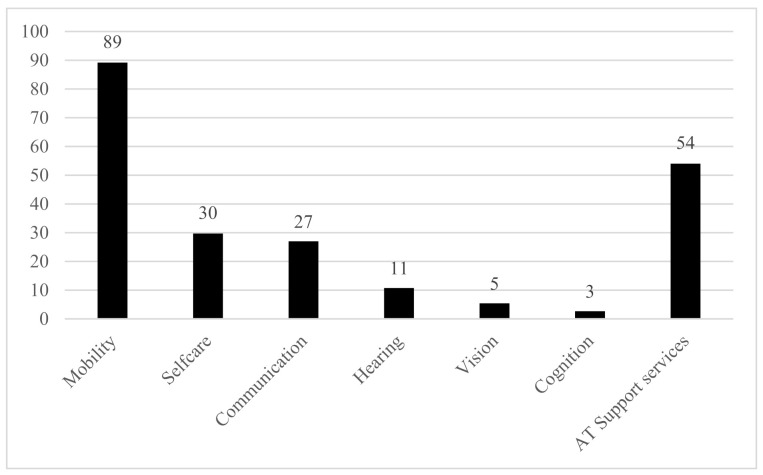
Percentage of responses received classified by assistive technology type. Note some services provide more than one type of assistive technology.

Half of all AT providers who responded (51%) reported providing AT for a single functioning area (either mobility, self-care, communication, cognition, hearing, vision), almost a third provided AT for two functioning areas and 15% provided AT for more than three functional areas. The most common functioning area AT providers reported providing AT for was mobility (89%) followed by self-care (30%) and communication (27%), as displayed in Figure 2.

Note that the quantitative data presented in this and following sections describe the experience of providers in the sample who are supplying this information and are indicative of the impact of COVID-19 on these AT providers. The data provide important context to the qualitative responses but are not intended to represent a global estimate of AT provision over the period.

### 3.2. Months Where AT Service Provision Was Impacted by COVID-19

**Figure 3:**
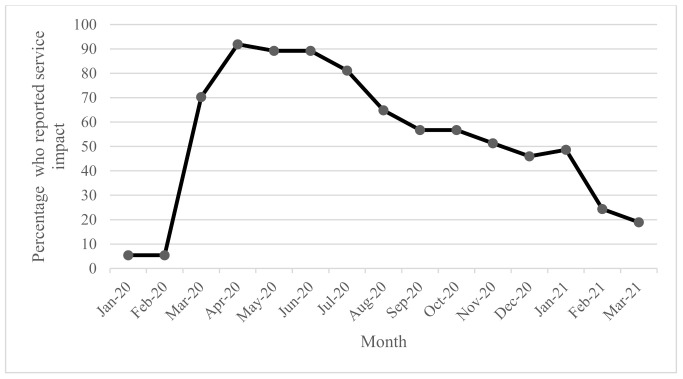
Percentage of responses that reported AT service impacts due to COVID-19 each month from January 2020 to March 2021.

Data were collected about the impact of COVID-19 on AT providers over a 14-month period from January 2020 to March 2021. Few AT providers (5%) reported impact on services in January and February of 2020 (Figure 3), but almost unanimously (92%) reported impacts on services in April or May 2020 (89%), June 2021 (89%), and July (81%). Impacts began to ease in August (65%), September and October (52%). Between November 2020 and January 2021, approximately half of all providers reported ongoing impacts, which eased again in February 2021 (24%) and further in March 2021 (19%).

### 3.3. Attendance at AT Services in 2020 Compared to 2019

**Figure 4:**
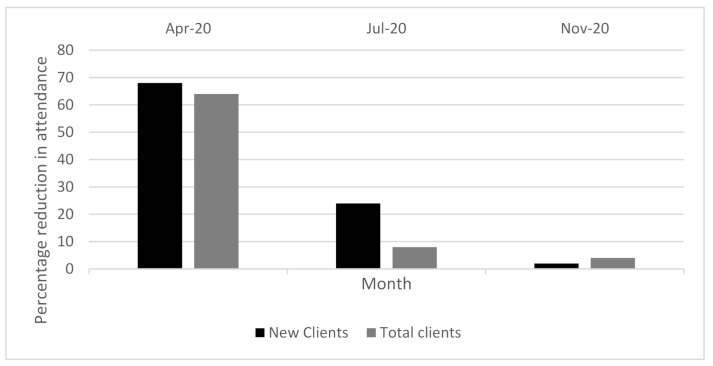
Percentage reduction in AT service attendance in 2020 compared to 2019 for new clients and total clients.

Client attendance was captured at three comparative time points: April 2019 and 2020, July 2019 and 2020 and November 2019 and 2020 (Figure 4). AT providers reported a significant drop in client attendance in April 2020 compared to April 2019 for both existing and new clients across all service types (mobility, self-care, communication, hearing and cognition, vision). Half of the AT providers reported a 100% reduction in attendance, reporting zero clients in April 2020. The average reduction in service attendance for April was 68% compared to April 2019. July saw attendance start to increase for new client attendance, but overall attendance remained well below 2019 numbers reduced by 24%, and overall client attendance was reported as reduced by 8%. Increasing attendance continued in November with client attendance almost returning to 2019 levels, noting a reduction of 2% for new clients, and 4% overall. No difference was noted between service types and attendance, but this should be interpreted with caution due to the low numbers of responses from some service types, for example, cognition and vision.

### 3.4. Factors That Impacted AT Service Delivery

**Figure 5:**
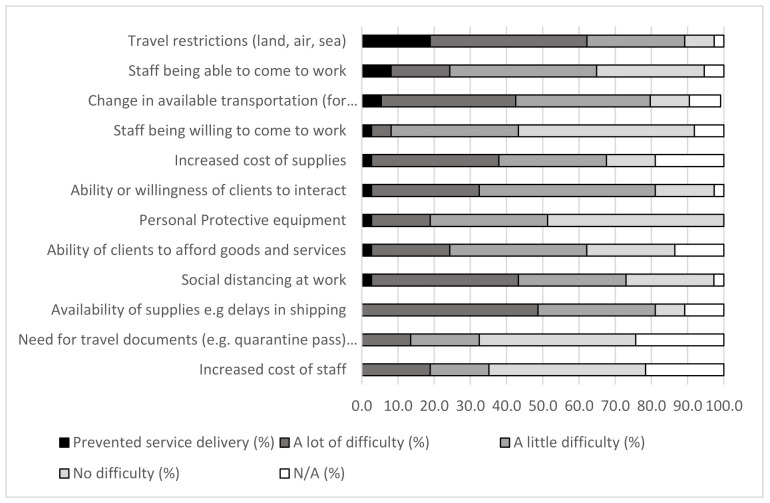
COVID-19 related impacts on assistive technology providers.

The COVID-19-related factor that had the biggest impact on AT service delivery was travel restrictions, which impacted 33 respondents (89%) and completely prevented service delivery in 7 respondents (19%) (Figure 5). The second greatest impact was the availability of supplies, which caused difficulty (a little or a lot) for 30 respondents (81%). Related to supplies as well, increased cost of supplies was reported as causing difficulty for 25 respondents (68%). Social distancing at work caused difficulty for 27 respondents (73%). 

Despite most respondents reporting a little or no impact of personal protective equipment (PPE) (81%), staff being able to come to work (70%), and the ability or willingness of clients to interact (64%), these factors prevented AT service delivery in a small number of responses.

### 3.5. Factors That Helped AT Providers during COVID-19

**Figure 6:**
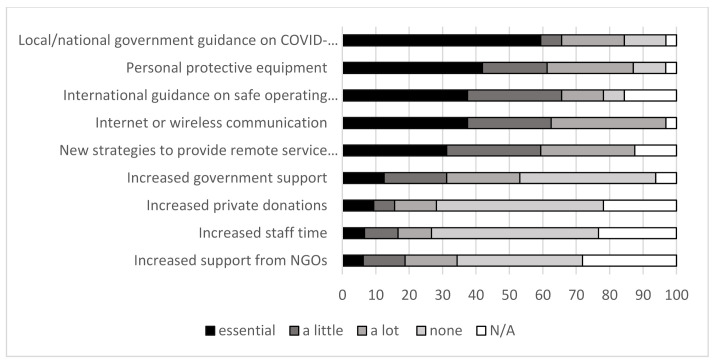
Things that assisted in responding to COVID-19 to maintain assistive technology services.

When asked to what extent factors helped AT services during COVID-19, respondents reported that the most helpful factors were: the internet or wireless communication, which was important in 32 respondents (97%), and local/national governmental guidance on COVID-19 safe health services, which was seen as essential by 59% of respondents and helped a lot in a further 19% of responses. Almost all respondents (89%) reported that new strategies to provide remote services was helpful. A little over half of respondents said that increased government support was helpful (Figure 6).

### 3.6. Thematic Analysis

**Figure 7:**
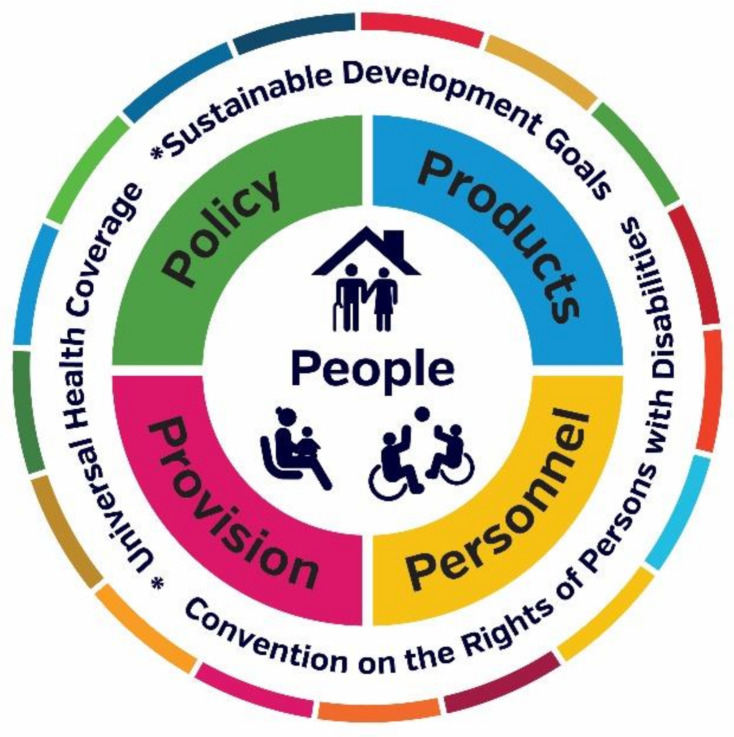
WHO’s 5P model for AT systems strengthening.

WHO describes a ‘5P’ model for AT systems strengthening that includes five key intersecting dimensions, comprising people (those who use AT), AT provision systems, AT personnel (providers) who supply assistive products and the underpinning policy [5] (Figure 7). The five elements of the AT ecosystem were used to apply a systems lens to the qualitative narrative. In the following section, themes are described using first-person illustrative quotes, which are identified by the country of response.

#### 3.6.1. Policy

The policy landscape presented similarities globally, across contexts; lockdown, or a reduction in the permitted movement of citizens, was almost universal. Respondents maintained that government-mandated lockdowns made it challenging and, at times, impossible for clients to attend their AT service.

*We were on lockdown declared by the Government. Lockdown prevented them [providers] from reaching cities where they could get these [hearing aids] sorted. So, we could not enrol them [new clients] without them using hearing aids or Cochlear implants*.

(SP01, India)

…*some of them cannot work due to the lock downs*.

(SP01, Columbia)

In some instances, respondents were positive regarding the action taken by governments to support the resumption of AT services following mandated lockdowns.

*The coordination with Ministry of Social Development help us in arranging the essential PPE for the staffs during the Lockdown Period (under 1 months). It helped us to resume the service after 1 month even lockdown period was challenging.*

(SP02, Nepal)

In instances across regions, respondents were critical of the government’s response to place restrictions on AT services and for categorising them as a ‘non-essential’ service in the public health response.

*The entire healthcare intervention initiated by the state could have been done in a different approach and could have been much inclusive. The panic created by the pandemic situation and social response to it created a difficult situation of noncooperation, mistrust, miscommunication among people, which led to ignorance of the regular needs of persons and children with disabilities significantly. We witnessed associated issues with adults and children with disabilities when they could manage to reach us, or we could reach out to them. The state could always have kept their priorities of the pandemic on the high but not closing down the health and rehab care provision largely*.

(SP01, Afghanistan)

Others were even more critical, suggesting that preventing people from accessing AT services placed them at risk of harm.

*Proposal of Protocols to let people come to AT services instead of saying nobody goes to AT services except urgencies. AT users in my context do not know what or when is urgent and they keep using some devices and got hurt (like orthosis or wheelchairs*).

(Argentina 01)

Providers were mindful of the effect of government policy on their clients, including the level of fear to attend services.

*Minor government laws e.g., lock downs, closing of AT provider’s facilities (shops, markets) due to shortage/delaying of orders from abroad and clients unawareness on matters relating to COVID-19, led to fear even to attend AT services*.

(SP01, Tanzania)

Government-mandated lockdowns also affected the sustainability of private AT businesses.

*COVID-19 pandemic definitely brings difficulties as an institution… we need to adjust especially the implemented policy based on the required health protocol which gives big influence on our Prosthetic Orthotic unit. The situation makes us have to close the services in the first initial 3 months (April–July) which then effects on the production load and operational cost. Due to the situation we decided to shift the production into face shield production. The aim is not only to give support to all of our assisted beneficiaries but also used as other income stream. The changes are not on our ability but on its effect for all of our clients to access services due to national policies of large scale social restriction, rapid test requirements for all clients as well as the shifting of almost all government budget for COVID-19 response which affect organization’s capacity as whole*.

(SP01 Indonesia)

*Minor government laws e.g., lock downs, Closing of AT provider’s facilities (shops, markets) due to shortage/delaying of orders from abroad and Clients unawareness on matters relating to COVID-19, led to fear even to attend AT services, unfortunately, we could only stop the service*.

(SP01, Tanzania)

Finally, weaknesses in pre-COVID-19 AT policy were more generally identified by some as having been put under the spotlight by COVID-19, highlighting areas for future improvements.

*The lack of a functioning AT Ecosystem in Ireland has become more ‘visible’ now to senior management in Enable Ireland and in the Health and Education sectors. The need for the establishment of such a system, beyond simply funding devices to tide people over during a time of crisis, is gaining more traction at present, and we are optimistic that with technology being front and centre for all, that we will finally see (and play a central role in) the establishment of such an AT Ecosystem nationally*.

(SP01, Ireland)

#### 3.6.2. People

Person-centred AT services place user needs, preference, and experience at the forefront of service delivery and decision making. AT providers expressed a strong motivation to maintain person-centred AT services throughout COVID-19. Person-centred communication was adopted by AT providers both related to delivering accurate and timely information and then being able to communicate service changes to clients as they occurred. Respondents described the innovative strategies they used to maintain communication with clients.

*Immediate set up of dedicated communication with the service users. Dedicated trained staff was assigned the roles to talk to the users and the families making them connected with us as service providers. Set up online platform to connect over video calls on virtual mode and started assessments and service deliveries, including therapy sessions, wheelchair services, positional devices, those which may not need much customization and measurement taking*.

(SP03, India)

*Radio messages, billboards, vehicle campaigns, follow up beneficiaries through a hotline*.

(SP02, Afghanistan)

*Provision of seminars to the client’s families on matters relating to COVID-19 so as to reduce fear*.

(SP01, Tanzania)

For this communication, respondents expressed that technology, in particular, smartphones, were especially important, as was tailoring the communication approach to the needs of the individual.

*For clients who did not have smart phones, we approached well-wishers who willingly donate smart phones. This was an effective manner of reaching out to the client*.

(SP03, India)

*Communicating with clients and service providers to ensure that urgent issues were being raised and addressed within COVID-19 guidelines and restrictions. Utilise phone, email and telehealth contact depending on the needs of the client and access to technology and history working with client. For example, clients well known to the service could be supported more easily* via *email and phone, whereas telehealth was more vital for new clients not known to the service*.

(SP01, Australia)

However, it was not always easy, with a lack of technology posing a barrier to communication.

*Lack of technology, lack of smart phones with the parents of the clients, intermittent internet facility, not clear internet connections were the challenges we faced*.

(SP01, India)

Finally, respondents noted that COVID-19 may have made services more person-centred by raising important conversations about access to AT.

*But all in all, the balance of power has shifted dramatically and many adults in particular are playing a much more active and equal role in service design than pre-COVID-19. COVID-19 has put access to technology into the centre of conversations about service provision*.

(SP01, Ireland)

#### 3.6.3. Provision

In countries where government policy allowed AT services to continue, respondents reported that they had to rapidly adapt their service delivery model to negotiate other barriers including transportation. Many respondents reported taking great measures to decentralise their service from an institution or hospital to people’s communities or homes.

*We quickly adopted the strategy to provide services through outreach in the community through home visits, meeting users in the clinics of the doctors and telerehabilitation through various platforms*.

(SP03, India)

*Change in transportation for clients they were not allowed to attend our hospital or to travel due to restrictions, so all assessments had to be at their home and these were restricted to urgent cases only*.

(SP01, Australia)

*We ensure product delivery to clients through courier services*.

(SP05, Bangladesh)

Where transport was a barrier to attendance, additional measures were taken by AT providers to assist both clients to attend their service setting:

*We began pick up and dropping off programs for our patients who needed surgery since lockdown affected movement by public means. It was effective since none of the patients scheduled for surgery missed out*.

(SP01, Kenya)

*We also made a decision in our AT Service to long term loan out devices formerly used for training, to adult service users, to enable them to get connected to the Virtual Service and to support their own social interactions outside of our service*.

(SP01, Ireland)

Telehealth, a service delivery model traditionally rarely used in AT services, was mentioned by only two responses, who noted difficulties with particular client groups and the need to further develop capacity in this area

*Clients not being able to see specialists so having to do this* *viatelehealth which is very difficult when clients are cognitively impaired and severely disabled*.

(SP03, Australia)

…*internet/technology access to be included in telehealth care and monitoring program. The biggest need was: Costs management, delivery of equipment/ supplies, technology to improve telehealth service (apps that are accessible to all, easy to use) and improve the quality of telehealth services*.

(SP02, Brazil)

Adjustments to services were not always seen as effective following reflection by AT providers:

*Assessment, fitting and delivery steps were compromised because of the patient’s fear of attending tests, exams, and consultations*.

(SP01, Brazil)

Furthermore, respondents from countries across income levels reported that upon undertaking rapid actions to adapt services, new challenges arose.

*We closed the facility, and we established home visiting program. This step was not real effective because we were not able to provide services to all our clients. Mobility from family to family led to extra use of resources like car fuels etc.*

(SP01, Tanzania)

*Changes to inpatient journey time frames (i.e., seeking more rapid discharge) resulted in additional difficulty/time pressure in meeting good seating outcomes*.

(SP02, Australia)

AT providers described how they changed staffing arrangements to manage social distancing to protect staff and clients within limited clinical spaces.

*decreased staff working daily*

(SP01, Morocco)

*optimization of teams and schedules to decrease contact time among patients, staff and transportation (bus, train, car, taxi, etc.)*

(SP02, Brazil)

*staff with high risk factors forced to work from home, other staff assisted with shortfalls or managed their patients*

(SP01, South Africa)

*During COVID pandemic most of Prosthetic Orthotic technicians assign to conduct night duty to reduce organization burden so that we do not have to use persons from outside our office*.

(SP01, Indonesia)

Given these adjustments, it is unsurprising that respondents, when asked what helped to maintain AT services, expressed that the flexibility and dedication of personnel were central in describing how services coped with the necessary changes:

*Willingness of staff to change timings to suit clients*

(SP01, India)

*Flexibility of patients, staff and company support to assist patients after hours (e.g., drop offs and collections, home visits)*

(SP01, South Africa)

*the motivation and dedication of staff helped a lot*

(SP02, Afghanistan)

Other service adjustments also related to safety.

*distribution of hygiene kits, masks and COVID-19 leaflets*

(SP03, Afghanistan)

#### 3.6.4. Products

The main concerns expressed by AT providers about products was the ability to safely and effectively support their clients to maintain their assistive products in the absence of being able to access a service and addressing issues of product affordability.

*Provided motivation to clients: teach how to maintain product safety*

(SP02, Bangladesh)

*What was the biggest need? Repair and maintenance of the assistive devices*

(SP03, India)

Given that more than half of respondents said that clients’ ability to afford their assistive products impacted their ability to provide service, it is unsurprising that they then went on to discuss their attempts to make assistive products more affordable.

*I had a preferential policy for loyal and new customers to encourage them to return to my store such as discounts and promotions… I choose suitable products to discount*.

(SP01, Vietnam)

Others suggested solutions to tackle this cost barrier,

*Tax policies that would make cost of fittings cheaper*

(SP01, Kenya)

When asked directly what the biggest help to services would be, many cited assistance with the cost of assistive products

*Low cost maintenance and support to rehabilitate their livelihood*

(SP04, Bangladesh)

*Reduce product cost, continuation of subsidy*

(SP02, Bangladesh)

*The support from government (funds, technological wise, exemption of taxes etc.). It is not done*

(SP01, Tanzania)

#### 3.6.5. Personnel

It is well known that there is a dramatic shortage of AT personnel globally, and this was highlighted by respondents from countries across all income levels who mentioned additional staffing capacity as the area of biggest need during COVID-19.

*More staff–recruitment and retention of staff is very difficult especially as we provide services in centres where the working population is very transient. More measures need to be put in place by Government to attract people to relocate to our territory, and to keep people here*.

(SP02, Australia)

*Available supportive fund/donation for human resource and raw material. Capacity building for staffs for new innovation*.

(SP01, Bangladesh)

*To have more staff, to get easily the supplies and to have a wide workspace so that all the staff can work daily*.

(SP01, Morocco)

*funds and human resources*

(SP02, Afghanistan)

AT provider networks were also mentioned as important in sharing good practice information between AT providers.

… *learning gleaned from our online CHAT (Community Hub for AT) gatherings where service providers shared good practice*… *We support local teams throughout the country to identify the most appropriate AT to meet their clients’ needs, in partnership with those clients and their families. This would not have happened, pre-COVID-19*.

(SP01, Ireland)

… *many providers did not have the information they needed. Our hospital ran out of hand sanitiser very quickly in the early days and we were told it wasn’t required and we had to wash hands. This wasn’t good in or outside of the hospital. Conflicting advice about the use of masks and the type of masks to use and face shields–feeling we were always behind what was happening*.

(SP01, Australia)

AT provider respondents offered positive impacts, which may influence future improvements in AT service delivery particularly related to the adoption of technology, which conflictingly presented an area of most benefit, but contrastingly most need. This was particularly well articulated by a respondent from Ireland:

*Overall, I think that COVID-19 has resulted in many positive impacts on our services, not least of which is the transition to online provision which circumvents the transport barriers which have previously curtailed so many activities. I anticipate that we will be keeping many of the practices we have adopted post COVID-19 and hopefully, reaching a far more diverse and larger cohort, who can avail of online services, with a reduction in the quantum of face to face delivery. However, this will require of us a continuous investment in IT infrastructure and an openness to trialing new models of delivery*.

(SP01, Ireland)

However, this positive envisioning is only feasible with foundation resources, as described below.

*Lack of technology, lack of smart phones with the parents of the clients, intermittent internet facility, not clear internet connections were the challenges we faced*.

(India 01)

… *internet/technology access to be included in telehealth care and monitoring program*.

(SP01, Brazil)

## 4. Discussion

Overall, the 5P model provided a comprehensive and nuanced way to delineate the impacts of COVID-19 response on AT service providers and their clients. The five elements captured thematic data exhaustively, and no outlying thematic areas were identified.

Policy was demonstrated to be a highly impactful dimension with unintended consequences for AT providers, identified as lockdowns and restrictions on movement resulted in service closure, attendance hesitancy/fear, and uncertainty. These resonate with calls in The Lancet for disability inclusive COVID-19 responses to address barriers of communication, physical distancing/isolation, and increased risk of infection [24].

Evidence demonstrates AT users experience accentuated health disparities due to their increased vulnerability to infection and inability to obtain routine supplies, including AT [25]. AT providers in this study demonstrated high awareness of these risks and attempted to address them, often despite blanket policy mandates, rapidly adapting their approach in attempts to maintain services. Some critiques are emerging regarding policy responses such as hard lockdowns and lack of disability inclusion [11,26]. There is much to learn with emerging recommendations supported by the data and the literature, focussing on how to design more inclusive pandemic response policy and reforming existing health policy.

An overriding theme within the domain of ‘people’ concerned person-centred services, with appropriate communication identified as essential to reach AT users. The diversity of communication strategies adopted by AT providers included various ‘occasions of service’, e.g., personalised contact from AT providers; regular telephone touch points; broader signage and advertising; through a range of media, e.g., online platforms; messaging via smartphone; social, print and audio media and vehicle campaigns. These findings resonate with a range of studies identifying the critical impact of communication during COVID-19, including recommendations by Lazarus et al., 2021 who suggest governments should make particular provisions to ensure all communications are accessible for people living with disability and those who use AT [27].

Emerging evidence demonstrates the AT user community across both high- and low-income countries, as a subset of the disability and ageing communities, experiences heightened anxiety and stress due to COVID-19 [16]. Additionally, this cohort are found to bear an additional burden related to stigma, specifically real concerns over rationing and priority-setting in the provision of needed resources, including AT [16,28,29,30]. Respondents in this research support this idea, with many raising concerns with how their clients feel about a range of issues not related to their AT, such as employment, financial security, food security and the ability to safely access transportation amongst others.

Successful provision of AT during COVID-19 pivoted on two pathways to access the service user, namely transport and virtual connection via telehealth. A substantial emerging literature suggests tele capability as a solution to service delivery [31,32], although noting some aspects of service such as physical examination and use of AT is difficult to replicate virtually. However, in our study, few respondents utilised telehealth, perhaps signalling barriers to uptake as a future area of enquiry. Further exploration of the experiences of AT users and families with telehealth can be found in related papers in this series [32,33].

Considering personnel, the voices of AT providers captured here present a nuanced picture of a highly dedicated workforce, known to be understaffed before COVID-19 [5,34]. AT provider respondents often reported demonstrating substantive and person-centred, culturally relevant services above and beyond what may be usually expected of an AT provider: sharing knowledge, adapting service models, flexibly creating workarounds to meet a changing healthcare landscape. These actions included delivering products to people’s homes, addressing clients’ financial barriers, helping clients source smart phones and many others. The highly dedicated approach garnered from participants in this research presented clear and deep concern for the wellbeing of AT users should they not be able to access their services. These perspectives align with many emerging recommendations and urgent calls to action in the current published literature [24,30] to provide guidance to assist and guide suitable service adjustments to be made across healthcare, rather than leaving providers to pave their own way.

Provision of assistive products and the wraparound services by AT providers was severely impacted by COVID-19. This picture is best understood through recent work by Smith et al. [2] who propose a range of strategies to develop inclusive and resilient systems in the face of COVID-19 for AT stakeholders. Smith and colleagues call for the championing of best practice service delivery models for robust and accessible remote service delivery, such as telehealth. Our data demonstrate global shortfalls in this respect. Supply lines for sourcing products and parts were disrupted. Service elements such as deployment were limited by lockdown and distancing requirements. Evaluation and fitting were often unable to be carried out face to face. Maintenance and repair of assistive products was disrupted, with a combination of AT user uncertainty and logistics affecting usual patterns of management. The repercussions of lost revenue for AT providers brought into sharp relief the perceived high costs of working with assistive products, with associated import, tax, and material costs. The empirical data discussed above reinforce the imperative to ensure countries develop sustainable infrastructure and policies for AT service delivery under rights-based frameworks.

This position paper on AT personnel [33] draws the above discussion threads together and acknowledges system complexity (through a matrix approach such as the 5P) as well as the importance of including AT users as central stakeholders in all planning strategies, policy reforms and public health responses. This approach will hopefully ensure that AT services are appropriately recognised by policy as essential, as considered by those who need them. It will also act to encourage all relevant actors to implement accessible and affordable approaches and tools in the event of a future global health crisis.

## 5. Recommendations

The recommendations described below were developed from the findings of this study and the studies of AT user experience reported in two sister articles [34,35]. The recommendations call for more inclusive public health responses, which recognise AT as essential products and services, and highlight that existing AT systems require strengthening to be better prepared for future challenges.

**The first recommendation is to make public health responses inclusive of people who use AT**. To do so, consultation with AT stakeholders is critical, including civil society, AT users, their families and representative bodies. Understanding how AT users are impacted by public health responses will enable mitigation strategies to ensure these responses are inclusive. AT-inclusive public health communication is required in multiple formats (for example, captioned; screen-reader friendly; plain language). Strategies for public health information to reach AT users, for example, the provision of information and communication technologies such as smart phones, must be considered.

**The second recommendation is to recognise AT as essential health products and services during a pandemic or health emergency.** This recognition is important to ensure that AT services remain open, safe and accessible alongside other essential health services. It also ensures procurement pathways for assistive products are prioritised and maintained alongside other essential health products such as medicines. To best understand what is needed to maintain safe AT services, public health response planning must include AT providers. Keeping AT providers and their clients safe during face-to-face service provision can be promoted by ensuring that AT providers are trained in methods of infection control, including the use of PPE. Acknowledging that during a pandemic, face-to-face services are not always safe, AT providers require capacity in provision of services via remote methods such as telehealth.

**Figure 8:**
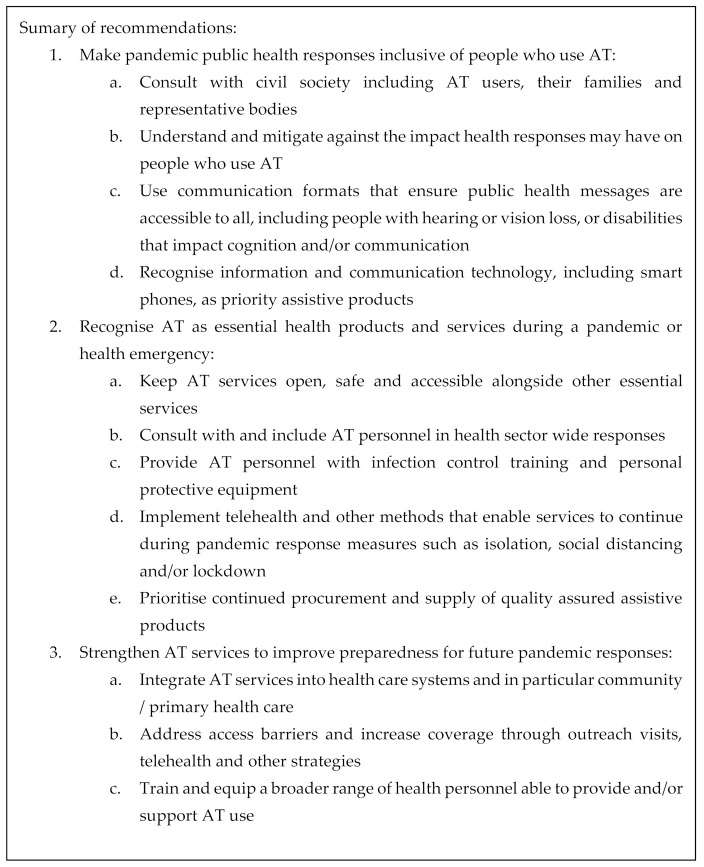
Summary of recommendations.

**The third recommendation is to strengthen AT services to improve preparedness for future pandemic responses**. AT services would be strengthened considerably by being integrated into health care systems, in particular primary and community healthcare. The implementation of outreach services and telehealth would also address the poor dispersal of AT services by increasing geographical coverage. Training and equipping a broader range of health personnel as AT providers is another mechanism to strengthen AT services. These recommendations are summarised in Figure 8.

## 6. Limitations

There are several important limitations to this research to acknowledge.

Firstly, the influence of biases should be considered. The nature of the data from a non-random (or non-probability) sample must be considered and an awareness of potential self-selection and self-reporting biases in the findings is important. Whilst self-selection and self-reporting produced valid data through directly engaging with organisations, and giving them a voice, it carried the potential for social desirability bias. Social desirability bias may have involved the over-reporting of positive results by organisations, especially if the positive results were seen as favourable to the WHO. Conversely, under-reporting of positive results may also have arisen, if negative results were seen as potentially leveraging further support from the WHO. However, the survey was a ‘self-administered’ method of data collection, which could decrease the prevalence of social desirability bias as the absence of the interviewer reduces the fear of receiving a negative evaluation.

Moreover, given the snapshot survey was presented only in English, there is a risk of misunderstandings of the questions posed, arising from language use and/or cultural differences despite the integration of a global research team and piloting of the survey across world regions to mitigate this. As the survey was only delivered in a written format, it did not allow for any misunderstandings to be resolved or clarifications to be sought to allow for real-time re-wording or explanation.

Additionally, the recruitment method did not allow for precise response rates to be reported. Furthermore, whilst responses were received from each of the six WHO world regions, some regions were more represented than others. Given there were only 37 responses from 18 countries, further research is required, and the results of this research should be considered in light of this small sample size.

Whilst providers of different AT service types were included in this research, the sample does not allow for comparisons to be made between different service types due to the low response rates from certain service types (e.g., communication). Further research is required to explore whether different AT providers were impacted differently by COVID-19.

## 7. Conclusions

Barriers to access AT existed well before COVID-19. These are known to include inadequate recognition for AT in policy, insufficient provision systems, a lack of personnel and low numbers of affordable high-quality assistive products.

COVID-19 placed health systems and health services including AT under pressure, with public health responses taking never before seen actions. Societal lockdowns and limitations of movement, changes to transportation, and increased fear and stigma are just a few of the results of this. The public health response to COVID-19 further revealed and exacerbated existing weaknesses in AT systems and services and posed some new challenges.

Challenges were met creatively and with dedication by AT providers who were innovative, using their ingenuity to make rapid adjustments to services in attempts to maintain services to their clients. Securing mechanisms of ongoing maintenance and repair for AT posed particular challenges.

To address challenges, infection control capability including the use of PPE is highlighted by this research as an area in need of urgent attention for providers of AT who come into contact with vulnerable populations, including older people and people with disability. Furthermore, capacity in the use of digital technology, remote service delivery and telehealth were all areas identified as in need of improvement.

The recognition of AT as essential health products and services as highlighted in the recommendations would ensure that AT providers are better supported throughout future global health crises. Areas that require additional support include safe ways to remain open and increased infection control capability including the use of PPE. Furthermore, capacity in the use of digital technology, remote service delivery and telehealth were all areas identified as in need of support.

## Figures and Tables

**Table 1:** Number of responses classified by income and region.

Income Classification	Responses Number, %	African Region	Region of the Americas	Southeast Asian Region	European Region	Eastern Mediterranean Region	Western Pacific Region
High Income	8 (21%)		United States of America 1	Singapore 2			Australia 4
					Ireland 1		

Upper–middle income	6 (16%)	South Africa 1	Argentina 1			
			Brazil 2 Colombia 1				China 1
Low–middle income	18 (49%)	Kenya 1		Bangladesh 5			Nepal 2
		Morocco 2		India 4			Vietnam 1
		Tanzania 2		Indonesia 1			
Low income	5 (14%)					Afghanistan 5	

TOTAL	37	6 (16%)	5 (14%)	12 (32%)	1 (3%)	5 (14%)	8 (22%)

## Data Availability

All relevant data have been supplied in the article and Supplementary Material. Individual responses received have been stored securely as per ethical approval.

## Appendix A

**Table A1:** Summarised survey question set.

Section	Prompts
Service information	Country Setting (city, urban, rural) Does the service provide outreach to other settings? How is the service funded?
AT service delivery during COVID-19	Which months was AT service impacted by COVID-19? Client numbers per month in 2019 and 2020
Impacts on service delivery	What factors caused difficulty in your ability to provide AT services? (no difficulty, a little difficulty, a lot of difficulty, prevented service delivery, not applicable) Personal protective equipment Social distancing at work Staff being able to come to work Staff being willing to come to work Availability of supplies e.g., delays in shipping Ability or willingness of clients to interact Travel restrictions (land, air, sea) Change in available transportation (for employees or clients) Need for travel documents (e.g., quarantine pass) to get to the facility Increased cost of supplies Increased cost of staff Ability of clients to afford goods and services Did other factors cause difficulty?
Factors that may have helped in providing services	Did any factors help you in providing AT services? (none, a little, a lot, essential, not applicable) Increased private donations Increased government support Increased support from NGOs Personal protective equipment Increased staff time Internet or wireless communication Local/national government guidance on COVID-19 safe health services International guidance on safe operating procedures (e.g., WHO guidance on COVID-safe health services) New strategies to provide remote service provision (describe) Did any other factors help you to provide services?
Ability to provide services	Did your ability to provide services change (not at all, a little, a lot, unable to provide services, not applicable) What steps did you take to overcome the difficulties? What steps did other entities like the government or civil society organizations take to help you and was their help effective? What is the biggest need of AT users currently? What are the biggest needs for AT service providers? What do you think would have been helpful but was not done?
